# Green Alternatives to Zinc Dialkyldithiophosphates: Vanadium Oxide-Based Additives

**DOI:** 10.1021/acsaenm.3c00425

**Published:** 2023-10-31

**Authors:** Andrew. J. Straiton, Thokozile. A. Kathyola, Callum. Sweeney, James D. Parish, Elizabeth A. Willneff, Sven. L. M. Schroeder, Ardian Morina, Anne Neville, Joshua J. Smith, Andrew L. Johnson

**Affiliations:** † Department of Chemistry, University of Bath, Claverton Down, Bath BA2 7AY, U.K.; ‡ School of Chemical and Process Engineering, University of Leeds, Woodhouse Lane, Leeds LS2 9JT, U.K.; § Diamond Light Source, Harwell Science and Innovation Campus, Fermi Ave, Didcot OX11 0DE, U.K.; ∥ School of Mechanical Engineering, University of Leeds, Woodhouse Lane, Leeds LS2 9JT, U.K.; ⊥ Infineum UK Ltd., Milton Hill Business and Technology Centre, Abingdon, Oxfordshire OX13 6BB, U.K.; # School of Design, University of Leeds, Woodhouse Lane, Leeds LS2 9JT, U.K.

**Keywords:** antiwear additives, green alternatives, metal oxides, vanadium, titanium, zirconium, molecular structures

## Abstract

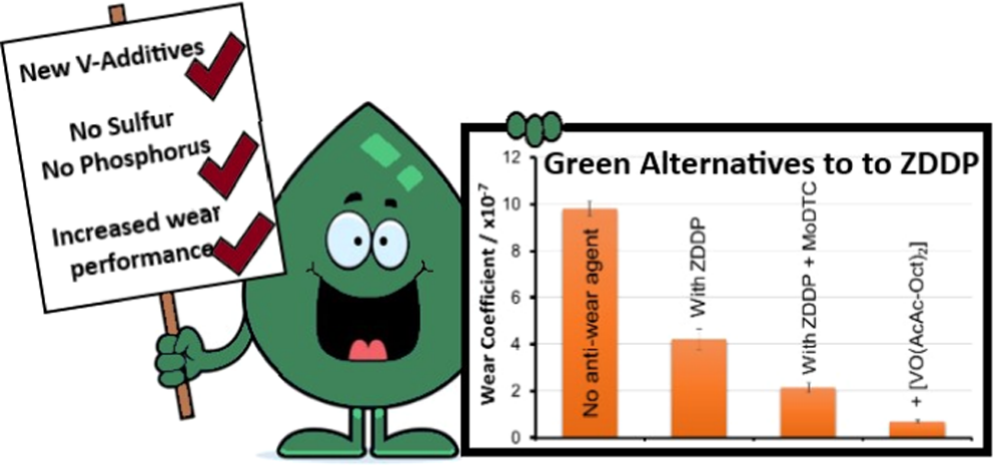

A functionalized vanadyl(IV) acetylacetonate (acac) complex has been found to be a superior and highly effective antiwear agent, affording remarkable wear protection, compared to the current industry standard, zinc dialkyldithiophosphates (ZDDPs). Analysis of vanadium speciation and the depth profile of the active tribofilms by a combination of X-ray absorption near-edge structure (XANES), X-ray photoelectron spectroscopy (XPS), and near-edge X-ray absorption fine structure (NEXAFS) analyses indicated a mixed-valence oxide composite, comprising V(III), V(IV), and V(V) species. A marked difference in composition between the bulk and the surfaces of the tribofilms was found. The vanadyl(VI) acac precursor has the potential to reduce or even replace ZDDP, which would represent a paradigm shift in the antiwear agent design. A major benefit relative to ZDDPs is the absence of S and P moieties, eliminating the potential for forming noxious and environmentally harmful byproducts of these elements.

## Introduction

The transportation industry accounts for approximately 19% of the world’s energy consumption and 23% of total greenhouse gas emissions.^1^ While the adoption of electric cars is ongoing, no timelines have been agreed for heavy-duty diesel (truck, plant, marine, etc.) engines, for which electrification and the adoption of greener technologies will take longer. As such, there is extensive ongoing research aiming to optimize every component of vehicles in order to reduce emissions.^2^ This includes the development of new low-viscosity lubricants that increase engine efficiency by lowering friction and contain lower levels of sulfur (S) and phosphorus (P), which significantly impair the performance of catalytic converters. However, the use of low-viscosity lubricants results in more sliding contacts operating in the boundary lubrication regime, with asperity contact increasing mechanical wear.^3,4^ The inclusion of effective antiwear agents is, therefore, an increasingly important element of lubricant design.

Currently, the most widely used antiwear agent in lubricants is zinc dialkyldithiophosphates (ZDDPs), which have become ubiquitous since their initial development in the 1940s.^5,6^ The protective, malleable phosphate glass formed *in situ* by the decomposition of these compounds is known to be a highly effective antiwear agent providing protection to engine components.^7,8^ However, sulfur and phosphorus compounds formed as byproducts are known to block active sites in catalytic convertors, impacting their ability to reduce exhaust emissions.^9−12^ The development of new low-S and low-P replacements to ZDDP is, therefore, a high priority not only in the transportation industry but also for other applications, such as energy generation and manufacturing, for which high-performance lubrication is required.^13−15^

It is estimated that through advances in tribological technology efficiency, savings of 40% can be achieved within 15 years, affording an 8.7% reduction in total global energy usage.^16,17^ This results in a drop in CO_2_ emissions of 3,140 MtCO_2_ (2733 and 406 Mt from friction and wear, respectively). Green tribology is, therefore, an important subfield of sustainable chemistry that is important to fully explore.^18^

Extensive materials research has identified a plethora of low-friction and wear-resistant coatings for automotive applications.^19−21^ However, with the exception of nanoparticle dispersions, a limited number of studies have sought to deploy prospective materials via the use of molecular additives in oil formulations.^22^ The design and implementation of oil-soluble molecular precursors offer a direct replacement for ZDDP, adopting the same mode of action by *in situ* formation of protective solid coatings between engine parts. Furthermore, with astute manipulation of precursor properties, decomposition to afford tribologically active films can be tailored to occur within specific engine conditions—for example, in areas where harsher conditions are experienced. Possible examples include selective action in the piston chamber, valve train, or in areas where protection from other components within the oil is limited.

**Scheme 1:**
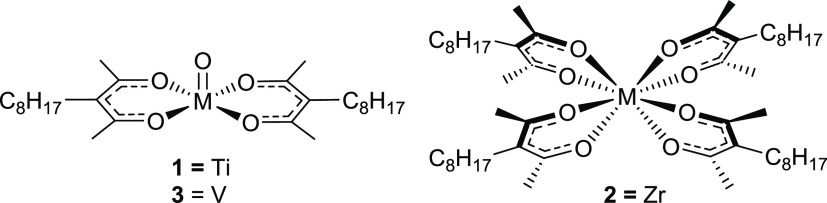
General Structures of Metal Acetylacetonate Complexes of the Type MOL_2_ and ML_4_

Building on the precedent of metal oxide deposition in the coating industry, we have targeted oxides of zirconium, titanium, and vanadium, which cover a range of tribologically interesting properties, for example, hardness and high melting points (ZrO_2_, TiO_2_) alongside lower melting points and potential lubricity afforded by a lamellar crystallographic structure with two-dimensional (2D) shear planes (V_2_O_5_). Acetylacetonate complexes of the three transition metals were selected as target precursor systems, as their chemistry is well-known, with longstanding applications in thermal deposition processes.^23−28^ Here, we detail the design, chemical characterization, and tribological assessment of three oil-soluble transition metal acetylacetonate (acac) additives (a general structure is shown in Scheme 1) forming solid films within the lubricated contact. We contrast their performance with current ZDDP technology through a variety of tribological tests. The resulting tribofilms are extensively characterized, and, based on these results, we present a mechanistic hypothesis for their effectiveness in reducing mechanical wear.

## Results and Discussion

**Figure 1:**
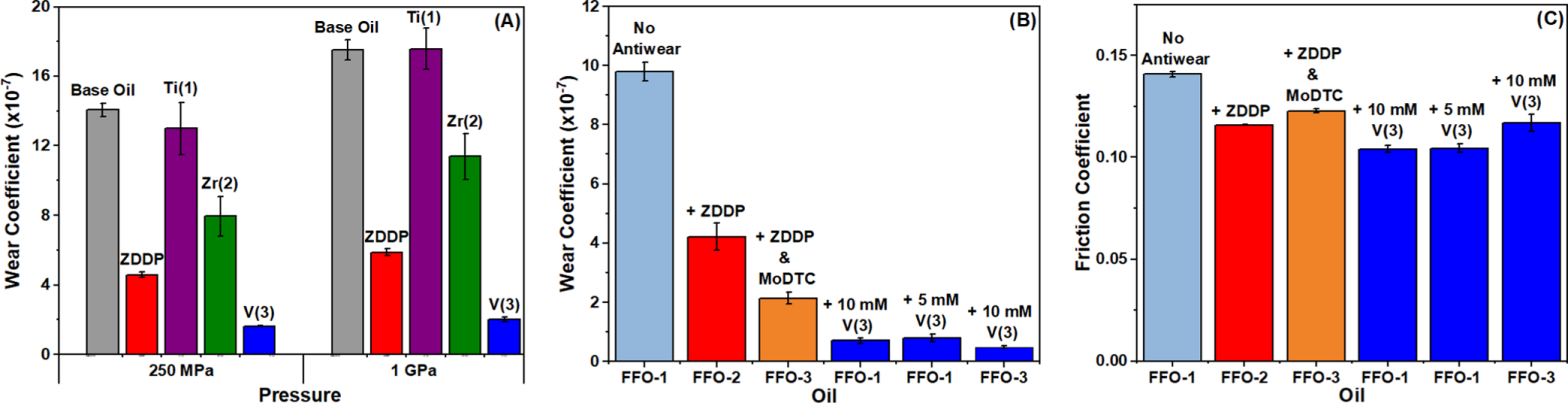
Wear behavior of complexes **1**–**3** dissolved in the base mineral oil (A), wear behavior of **3** in commercially representative model oils (B), and friction behavior of **3** in commercially representative model oils (C). All tribological tests were run at 100 °C and 1 GPa. Details of the model oils used are given in Table S2.

Engine components are typically subjected to a variety of harsh conditions during operation, so any viable replacement for ZDDP must withstand high-pressure regimes in which ZDDP excels and for which suitable replacements have not yet been identified. Figure 1a presents wear data obtained using formulations of the titanium (**1**), zirconium (**2**), and vanadium (**3**) compounds in mineral oil at concentrations commensurate with standard ZDDP loading (10 mM). The solid-state molecular structures of complexes **1** and **3**, as determined by single-crystal X-ray diffraction studies, are included in the Supporting Information (Figure S1).

Good solubility in the base mineral oil was observed for all three compounds to concentrations of 50 mM, with no sedimentation observed over a period of 7 days. The addition of zirconium (**2**) and vanadium (**3**) additives results in significant reductions in wear at both contact pressures (Zr, 44% at 250 MPa, 35% at 1 GPa; V 88% at 250 MPa, 89% at 1 GPa), with the vanadium compound (**3**) demonstrating the greatest wear protection. Tests at multiple concentrations (1–50 mM) show a significant reduction in wear can be observed upon the addition of compound **3** at concentrations as low as 1 mM (Figure S3, 250 MPa, and Figure S4, 1 GPa). No change in wear activity was observed with the addition of the titanium formulation (**1**). Friction coefficients recorded upon the addition of compounds **2** and **3** show a small increase, comparable to that typically observed upon the addition of ZDDP (Figures S5 and S6).^29^ A more significant increase in friction coefficient is observed upon the addition of the titanium compound, **1**.

**Figure 2:**
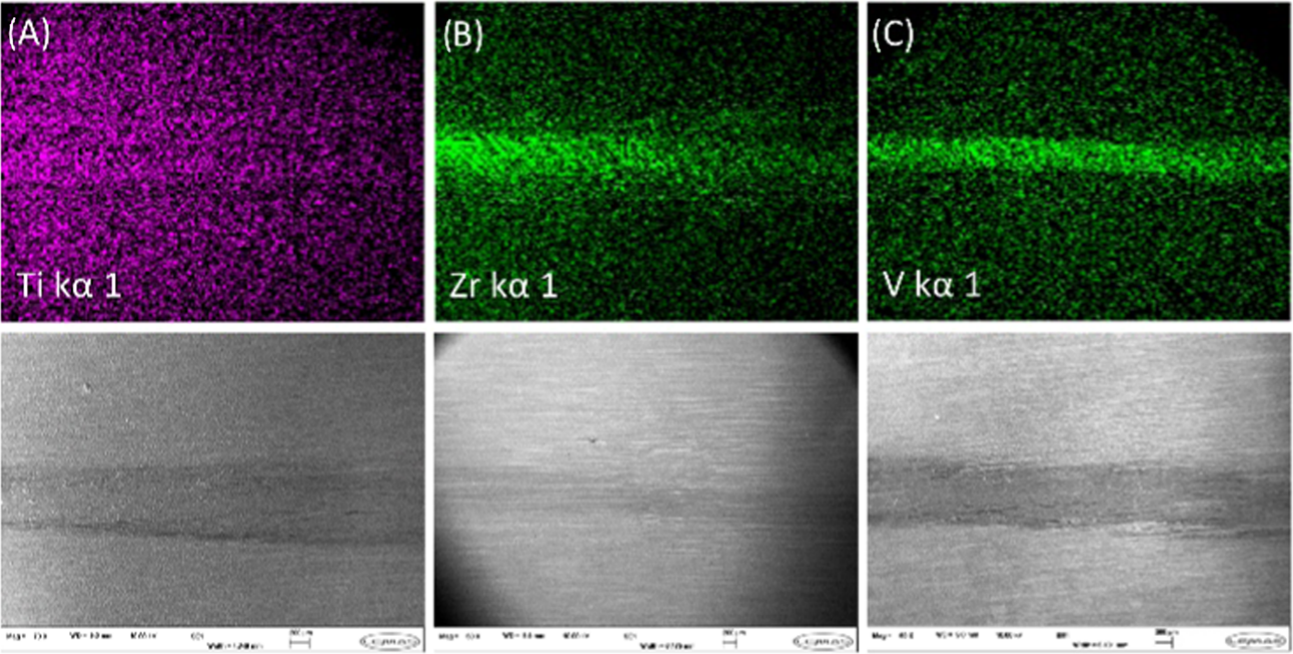
SEM–EDX mapping images of relevant elements for each tribofilm deposited within the wear scars. Substrates were obtained from tribological tests in the base mineral oil containing 10 mM concentrations of **1** (A), **2** (B), and **3** (C).

In each case, the formation of the respective metal oxide film was confirmed via scanning electron microscopy (SEM) and energy-dispersive X-ray (EDX) spectroscopy. Figure 2 clearly shows the presence of deposited metal-containing films within the wear scar. The bright (high metal concentration) and dark (low metal concentration) regions of EDX pertain to the inside and outside of the wear scar, respectively. This confirmation attests to the tribological decomposition of each metal additive as intended and is indicative of the fact that the observed tribological performance of each additive is a function of the material formed. High-melting-point oxides of zirconium and titanium have previously drawn interest as hard, wear-resistant coatings for some time,^30−33^ with nanoparticle dispersions showing beneficial tribological effects in some studies.^22,34−37^ Different perspectives have been offered on the tribological activity of vanadium derivatives, with research identifying the lamellar nature of low melting, crystalline V_2_O_5_ as a means of enhancing lubricity via 2D shear planes within tribofilms.^38^ This mechanism would be analogous to the mode of action of the ubiquitous solid lubricant added to engine oils, MoS_2_.^4^ In contrast, VO_2_ is a hard high-melting-point material, with tribological properties more likely to resemble the oxides of titanium and zirconium.^39^ Other studies have sought to correlate the frictional response and lubricity of a variety of metal oxides to the ionic potentials of their constituent elements, identifying V_2_O_5_ as a promising low-friction coating.^40^ However, no assessment of performance as an antiwear coating has been reported to date.

While screening the efficacy of prospective additives in pure mineral oil systems provides a good indication of antiwear activity, any novel additive must also function effectively in a fully formulated commercial oil, a feature that is imperative for commercial adoption and often overlooked in a research context. High-performance lubricants are complex, multifunctional fluids containing multiple components capable of synergistic and antagonistic interactions with other additives. It is well-documented for ZDDP that antagonistic interactions necessitate higher additive loading, compounding detrimental effects on emissions.^41^ As such, an assessment of the most promising vanadium additive (**3**) was undertaken in a commercially representative fully formulated model engine oil (FFO-1, see Table S2). The results of the testing, along with relevant comparisons, are displayed in Figure 1b. Control studies of FFO-1, along with representative additions of ZDDP and a standard MoS_2_ precursor, molybdenum dithiocarbamate (MoDTC), were undertaken to provide an accurate and consistent comparison with current technology and commercial standards. These additions are cataloged in comparison studies using oils FFO-2 (FFO-1 + ZDDP) and FFO-3 (FFO-1 + ZDDP and MoDTC, representative of heavy-duty diesel applications). Full solubility of additives **1**–**3** in all fully formulated oils was observed, with no sedimentation observed after a period of 7 days in any case. Details of the model FFO specifications used within this study are given in Table S2.

As expected, a significant reduction in the wear coefficient was observed with the addition of ZDDP (FFO-2), an effect increased by the addition of MoDTC (FFO-3) in a well-documented synergistic interaction between the two ubiquitous additives.^42,43^ The addition of vanadium additive (**3**) to FFO-1 at 10 mM resulted in exceptional wear protection, with wear reduced by 93% cf. 57% upon the addition of ZDDP and 78% on the addition of ZDDP and MoDTC. In addition, the antiwear response was comparable when the V concentration was halved to 5 mM (Figure 1b). This represents superior performance relative to that of ZDDP additives. Moreover, an additional synergistic reduction in the wear coefficient was observed upon the addition of the vanadium additive to FFO-3, the commercially representative model formulation containing both ZDDP and MoDTC. Interestingly, this reduction in friction was not observed during the testing of compound **3** in the base mineral oil (Figures S5 and S6), making this an unexpected observation that, without further work, we are unable to explain confidently. These results suggest several strategies for the application of vanadium additives for wear reduction in commercial oils. Not only do the results suggest complete replacement of ZDDP is possible within the regimes studied, but decreased ZDDP loading may also be implemented.

It is important to note that despite the fundamental importance of protecting mechanical components from wear, the reduction of friction and relative increase in efficiency are equally critical. While the addition of ZDDP (FFO-1 vs. FFO-3) results in no measurable frictional response, the addition of the vanadium additive (**3**) to FFO-1 results in a drop in the coefficient of friction of 26% (Figure 1c). Addition of V to FFO-3 also results in a significant reduction in friction (5%). Such results indicate that as well as affording wear protection greater than that of ZDDP, vanadium-based tribofilms also offer a significant frictional benefit over current additive technology, demonstrating potential fuel economy benefits and further mitigation of environmental impact. Friction and wear coefficients recorded during testing at multiple concentrations and at two pressures show behavior commensurate with that described above (Figures S7–S10).

### Tribofilm Structure

**Figure 3:**
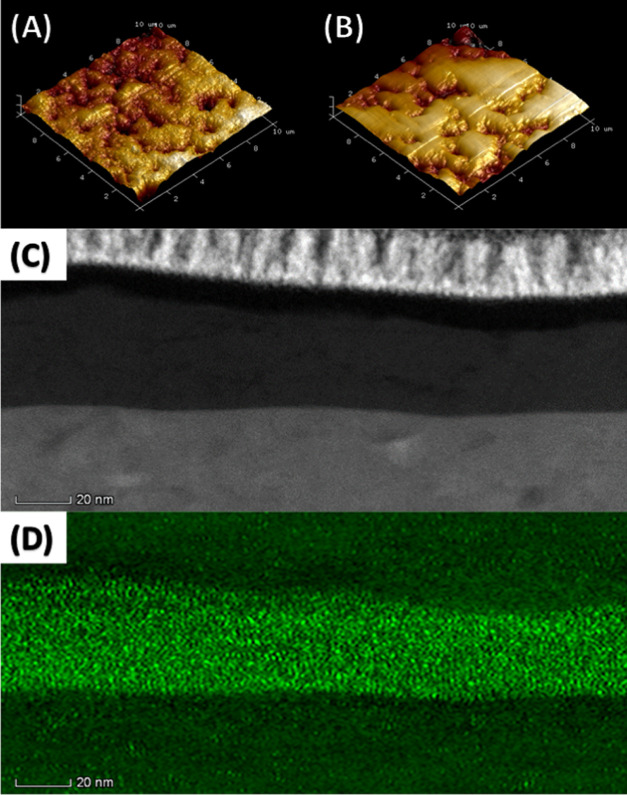
AFM images of tribofilms formed from **3** (A) and ZDDP (B). The cross-sectional TEM image of a tribofilm formed from **3** (C), with EDX demonstrating a high localized concentration of V within the tribofilm (D). Further EDX images are shown in Figure S15.

Given their ubiquity, zinc-containing phosphate tribofilms and their mode of action have been the subject of extensive study.^8,29,44^ Research suggests that wear protection is afforded by the formation of phosphate glass pads formed in tribological contacts,^45^ which are clearly observed in atomic force microscopy (AFM) imaging (Figure 3b). Consequently, research to develop a replacement technology has aimed to find ways of replicating these properties.^41,46^ However, AFM imaging of the vanadium oxide tribofilms reveals a morphology (Figure 3a) different from that of the ZDDP systems. Considered alongside the observed decrease in friction coefficient, this alludes to an alternative mode of antiwear action more commensurate with conventional protective coatings.^47^ Cross-sectional transmission electron microscopy (TEM) of the same tribofilm (Figure 3c) shows a 20 nm tribofilm composed of V (30%), O (48%), C (17%), and Fe (5%) as determined by EDX (Figures 3d and S11).

**Figure 4:**
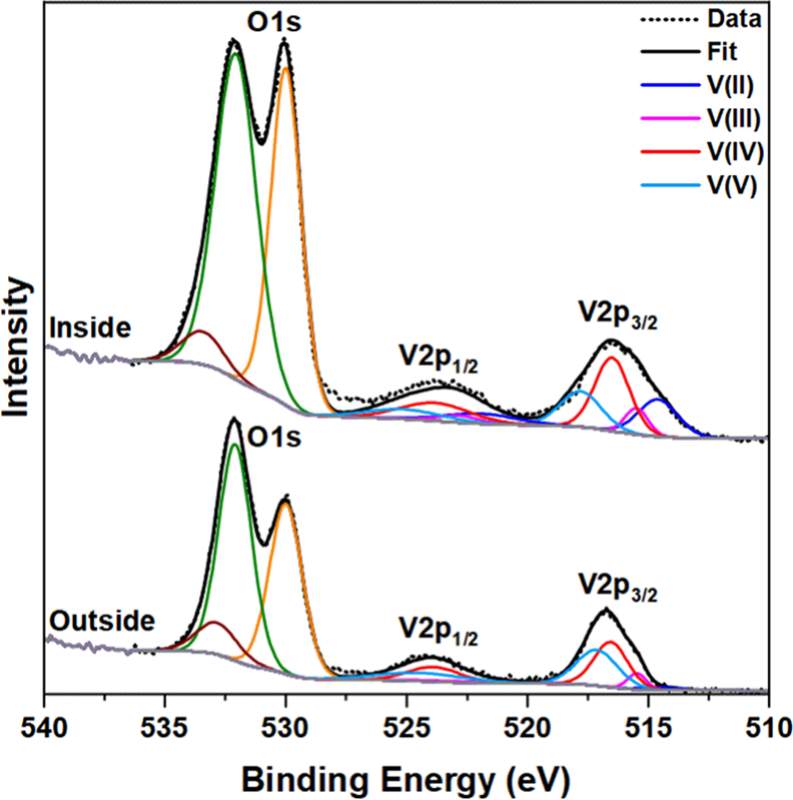
Fitted XPS spectra obtained inside and outside the wear scar following tribological testing of **3** in the base oil. Peak fitting data are given in Table S3.

While underexplored, various degrees of tribological activity have been reported for vanadium-based coatings,^39^ with investigations into vanadium nitride as a conventionally “hard” protective layer reporting lubricious activity upon *in situ* oxidation to V_2_O_5_.^38^ XPS analysis (Figure 4) of the tribofilm formed in our study underlines the complex nature of tribological properties, but also offers elucidation of both the materials present and a possible explanation for the exceptional tribological activity observed. Despite the delivery of tribofilms via a V(IV) additive, analysis of the V 2p_3/2_ emission suggests the presence of multiple vanadium oxidation states within the tribofilm, comprising V(II) (24%), V(III) (10%), V(IV) (42%), and V(V) (24%). While the reduction of V_2_O_5_ to VO_2_ under tribological and thermal stress has been reported previously, no such reduction of V(IV) materials has been reported previously.^48,49^ Residues outside the wear track (Figure 4) were found to comprise mostly V(V) (57%) and V(IV) (36%), alongside much less significant contributions from V(III) (4%) and V(II) (3%). This marked disparity (Figure S12) suggests that the reduction observed in the vanadium tribofilm occurs as a result of an effect within the tribological contact.

**Figure 5:**
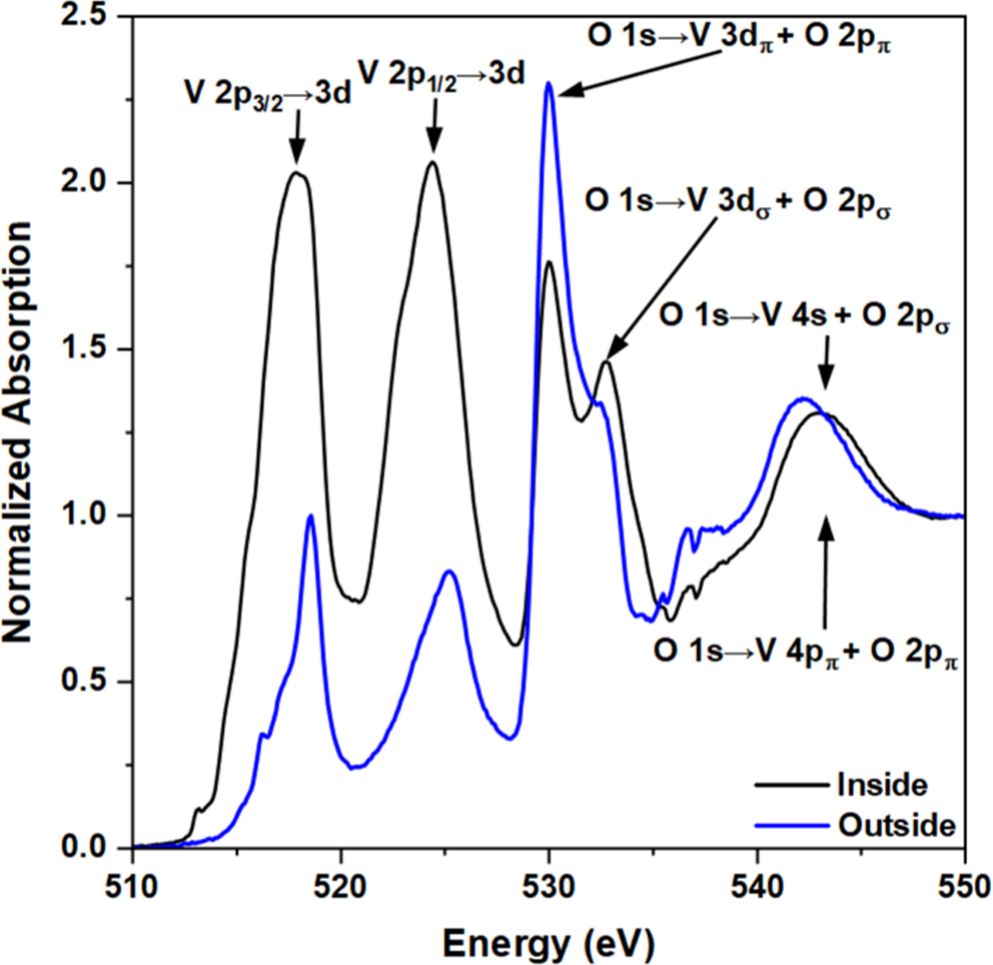
V L_2,3_-edge and O K-edge NEXAFS spectra obtained inside and outside the wear scar following tribological testing of an additive (**3**) in the base oil.

Further characterization by soft (NEXAFS) and hard (XANES) X-ray absorption spectroscopy (XAS) confirms the reduction of the vanadium species and the presence of multiple oxidation states in the tribofilm formed from **3**. The soft X-ray V L_2,3_-edge and O K-edge NEXAFS spectra obtained outside and inside the wear scar are shown in Figure 5. The two peaks at about 518 and 525 eV correspond to electronic transitions from V 2p_3/2_ (L_3_) and 2p_1/2_ (L_2_) core levels to unoccupied V 3d states, respectively.^50^ The remaining features are due to transitions from the occupied O 1s core level to unoccupied 2p states mixed with V 3d, 4s, and 4p states. Consistent with XPS (Figure 4), the intensities of the V 2p_3/2_ → 3d and V 2p_1/2_ → 3d peaks show that there is approximately twice as much vanadium inside the wear scar. The 0.8 eV red shift of these peaks confirms the reduction of vanadium from IV (outside) to predominantly III (inside).^50^ The reduction to V(III) is associated with higher occupation of 3d states, which is reflected by the limited availability of unoccupied states and, hence, a decrease in the intensity of the O 1s → V 3d_π_ + O 2p_π_ peak at 530 eV.^51^

The low resolution of the two V 2p → 3d peaks (Figure 5) makes it difficult to determine the presence of mixed valences. However, comparison with the spectra of vanadium oxides and vanadyl acetylacetonates allows identification of the species likely to be present, alongside inferences about the coordination geometry. The spectrum obtained outside the wear scar resembles that of VO(acac)_2_, whereas that obtained inside the wear scar is more similar to that of V_2_O_3_.^52−55^ Comparison of the peak shapes suggests that the species present outside and inside the wear scar have distorted square-pyramidal {VO_5_} and octahedral {VO_6_} moieties, respectively. This implies that even after exposure to high temperature, **3** retains the MOL_2_ structure (Figure 1) outside the wear scar—as would be expected, given thermal analysis does not show the onset of decomposition until ∼200 °C (Figure S2). However, inside the wear scar, the combination of high local temperatures and pressures results in the formation of the V(III) species. The O 2p ligand field splitting is also much more apparent inside the wear scar, suggesting the presence of a more structurally ordered vanadium species.^51^ Notably, the O 1s → V 3d + 2p features (Figure 5) become less distorted as the ligand site symmetry increases (square-pyramidal to octahedral). This variation in order is also evident in the V K-edge XANES.

**Figure 6:**
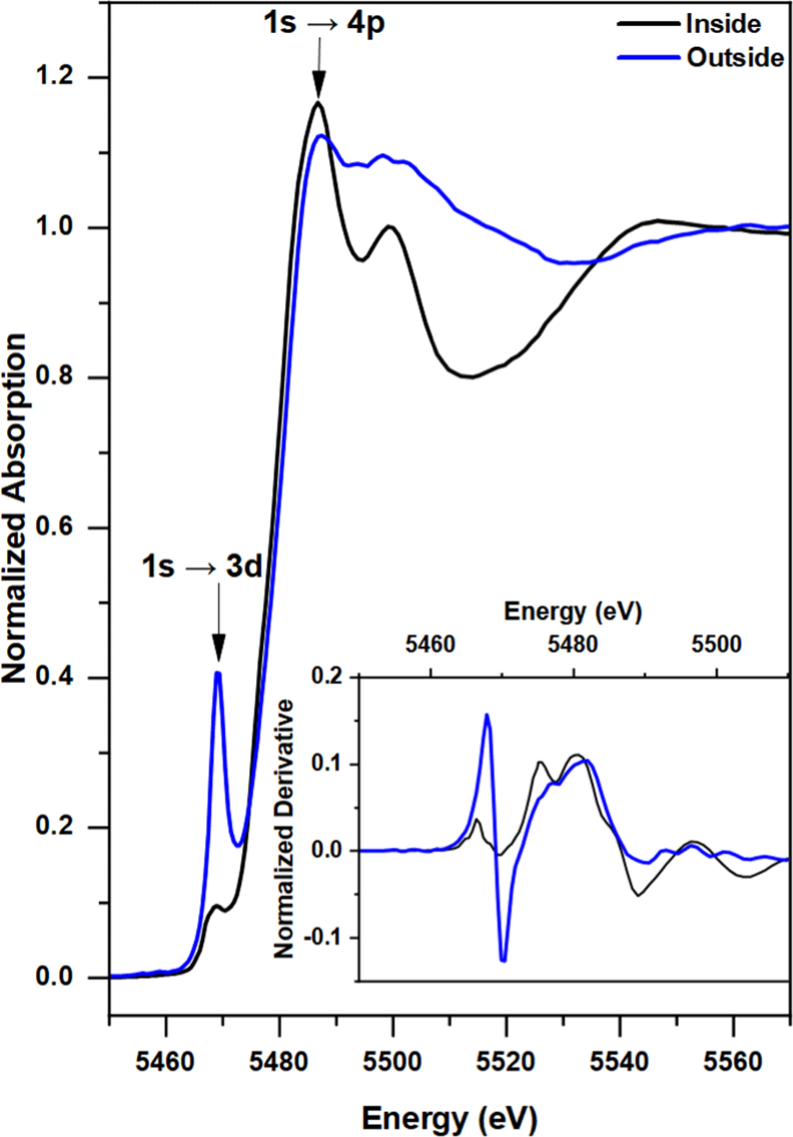
V K-edge XANES spectra obtained inside and outside the wear scar following tribological testing of **3** in the base oil.

Analysis of hard X-ray V K-edge XANES spectra again shows more V(IV) outside and predominantly V(III) inside the wear scar, indicated by a 0.8 eV red shift of the 1s → 3d peak (Figure 6), identical with the shift observed in the two V 2p → 3d NEXAFS peaks (Figure 5). A complementary X-ray fluorescence (XRF) map (Figure S13) distinguishes the inside and outside of the wear scar and shows the V concentration variations in the two regions. Much like XPS, XANES shows the presence of multiple oxidation states. A comparison with V_2_O_3_ and VO_2_ XANES spectra (Figures S14 and S16) shows that the species inside the wear scar is predominantly V(III); however, some V(IV) remains present. This is most evident in the derivative, where most of the spectral features inside the wear scar resemble those of V_2_O_3_, except for the second and third peaks. The second peak (absorption edge) is located between the peaks for V_2_O_3_ and VO_2_, and the shape of the third peak appears to be a mixture of the peaks for the two oxides. Comparing the vanadium oxide peak positions (Figure S14) suggests that the V species inside the wear scar has an average oxidation state of approximately 3.4. Conversely, the XANES derivative for the species outside the wear scar (Figure S13B) shows spectral features similar to those of both **3** and VO_2_, which have an oxidation state of 4, again suggesting that limited decomposition of **3** occurs outside the tribological contact at 100 °C. Notably, linear combination fitting of the spectra obtained outside and inside the wear scar was inconclusive. Further analysis of vanadium compounds beyond those reported in this paper is required. This composition differs from that observed via XPS, the more surface-sensitive technique, therefore demonstrating a difference between the surface and bulk of the tribofilm.

As discussed previously, symmetry and structural disorder have a significant effect on the XAS spectra of vanadium oxides. The pre-edge 1s → 3d peak in V K-edge XANES is reported to become more apparent with increasing static disorder and oxidation state.^54,56^ This peak is defined by the mixing of the V 3d and 4p orbitals combined with the overlap of the V 3d and O 2p orbitals. As can be seen in Figure 6, the peak intensity decreases from the outside to the inside of the wear scar as the symmetry of the vanadium center increases from distorted square-pyramidal to octahedral. Static disorder in the species outside the wear scar is also evidenced by the lack of a prominent peak at about 5500 eV. This postedge feature can be defined by various phenomena such as transitions to higher np states and/or shape resonances.^56^ However, in this case, it can be assumed that the difference between the two spectra is mainly due to multiple scattering, wherein the presence of acac ligands (with C and O scatterers) outside the wear scar suppresses the strong V–O multiple scattering contributions. This masking effect is also evident in XANES for **3** when compared to vanadium oxide standards (Figure S15).

Combining XPS, NEXAFS, and XANES suggests a remarkable difference in composition between the inside and outside of the wear scar, indicative of phase transitions due to tribochemical reactions in the wear scar. There are various possible causes of such transitions, but given the system being studied, it is hypothesized that they may be the result of either triboemission, a phenomenon whereby electron emission is induced within metal-based tribological contacts,^57^ or the amorphization of the V(IV) additive and subsequent crystallization of a V(III) species. Similar tribochemical transformations have been reported previously for V_2_O_5_ during milling.^58,59^

It is unlikely that the minor V(V) component (24%), attributed predominantly to V_2_O_5_, observed on the surface of the tribofilm accounts exclusively for the extraordinary degree of wear protection afforded by these tribofilms (Figure 1). We hypothesize that the lower melting point (600 °C)^60^ relative to other vanadium, zirconium, and titanium oxides, and the crystallographic lubricity of V_2_O_5_ plays an integral role in the frictional benefits and shearing of the material observed at the surface of the tribofilm, while the presence of harder, high-melting-point V(III) and V(IV) oxides in the bulk of the tribofilm (VO_2_—1500 °C, V_2_O_3_—1950 °C and V_6_O_13_—1500 °C)^61^ contribute significantly to the enhanced degree of wear protection afforded by these vanadium composites. Such activity has been suggested in previous studies on vanadium nitride coatings,^38^ and the application of composite coatings comprising both hard and lubricious materials has been previously reported.^62,63^ However, this study presents the first example of such a composite to be delivered and formed *in situ* from an oil-soluble and commercially viable single-source molecular precursor.

## Conclusions

We demonstrate here that a vanadyl(IV) acac system (**3**) suitable for use as a direct replacement for ZDDP affords remarkable wear protection in tribological testing. In addition, sulfur and phosphorus oxides originating from additives in the engine oil, such as ZDDP, have been shown to reduce catalyst effectiveness and block filters in exhausts. Therefore, it is particularly important to develop green antifriction and antiwear agents with excellent tribological properties and low sulfur and phosphorus levels, such as the vanadyl(I) acac complex (**3**) described here.

Characterization of the tribofilms suggests *in situ* tribochemical reduction to generate what appears to be a mixed-valence VO_*x*_ composite with V species akin to those in V_2_O_5_, VO_2_, and V_2_O_3_. The mechanism by which these materials affords wear protection is the subject of an ongoing investigation. However, initial tribological evaluation demonstrates that VO_*x*_ precursors have the clear potential to replace or minimize the use of ZDDP, affording potential access to next-generation environmentally friendly engine oils. Such development of next-generation antiwear agents replacing ZDDP will be essential for the minimization of the environmental impact of internal combustion engines.

## Methodology

### Metal Acetylacetonate Complexes

Titanyl (Ti^IV^O, **1**), zirconium (Zr^IV^, **2**), and vanadyl (V^IV^O, **3**) acetylacetonate (acac) complexes (general structures are shown in Scheme 1) were identified as stable and inexpensive compounds with the potential to form their respective metal oxides when subjected to thermal or tribological stress. The zirconium and vanadium analogues were synthesized in good yields via adapted literature procedures (Supporting Information) to yield hydrolytically stable yellow oils (**2**) and green crystals (**3**). The titanium-based formulation was derived from the reaction of titanium tetrakis-isopropoxide with two equivalents of C_8_-acacH, followed by stoichiometric hydrolysis with H_2_O, yielding a hydrolytically stable homogeneous yellow oil. ^1^H NMR studies showed that the product contained a mixture of isomers in line with literature precedent.^64^ Analytically pure materials can be obtained via crystallization from hexane (Supporting Information, SI). Thermogravimetric analysis (Figure S2) of all three complexes demonstrated sufficient thermal stability above 80 °C, which is necessary to avoid decomposition in the oil reservoir. Aliphatic chain substituents on the acetylacetonate ligand backbone ensured good solubility in the base mineral oil at room temperature for all systems to concentrations commensurate with typical ZDDP additive loading (∼10 mM).

### Tribological Studies

Initial tribological evaluations in the base mineral oil (Group III, 4 cSt) were conducted at 100 °C using a TE77 pin-on-plate tribometer, simulating boundary regime conditions (λ = 0.025), at contact pressures of 250 MPa and 1 GPa. All tests were conducted in triplicate to ensure repeatability. Both the pins and plates used were made of bearing steel with a Young’s Modulus of 210 GPa and a Brinell hardness of 7450 N/mm^2^. The steel plates had dimensions of 7 × 7 × 3 mm and a surface roughness of 0.1 μm *R*_a_. Testing was conducted for 1 h at a constant temperature of 100 °C, a sliding speed of 0.35 ms^–1^, and a pressure of 50 N. Pins with different curvature radii were used to generate the two different contact pressures reported. Such conditions result in thin lubricant films relative to the roughness of the surfaces, producing high levels of wear due to the degree of metal–metal contact (SI Section S1.4). The contact pressures used are consistent with those found in two wear-critical areas of an engine: the piston ring and liner contact and the valve train cam and follower contact. Worn volumes of the pins were measured using three-dimensional (3D) white light interferometry profilometry, using a noncontact NPFLEX, and converted into dimensionless wear coefficients via Archard’s eq (SI Section S1.5).

### SEM

The morphology and elemental composition of tribofilms formed from the testing of additives **1**, **2**, and **3** were studied using SEM and energy-dispersive X-ray (EDX) spectroscopy. Wear scars and tribofilms on the steel tribometer plates were analyzed using a Hitachi SU8230 microscope operated at 2.0 kV. Images were collected at a magnification of 50× and processed using ImageJ software.

### TEM

The morphology of tribofilms derived from additive **3** was further characterized using TEM. A lamella of the steel plate and tribofilm was prepared via the *in situ* lift-out method using an FEI Helios G4 CX DualBeam microscope. The lamella was attached, using an ion beam-deposited Pt layer, to a copper-focused ion beam lift-out TEM grid mounted within the SEM chamber and then stored under a vacuum before TEM analysis. Thinning and polishing of the lamella to electron translucency were performed with a final polish/clean with a gentle ion beam operated at 5 kV and 41 pA. All TEM measurements were performed on an FEI Titan^3^ Themis 300 operated at 300 kV. Bright-field TEM images were collected using the Gatan OneView 16 Megapixel CMOS digital camera using Digital Micrograph (GMS3) software. The TEM is fitted with the Super-X EDX system with a windowless 4-detector design. Elemental maps were collected at a probe current of 200 pA and a dwell time of 20 μs.

### AFM

Images of the additive **3** tribofilm on a steel plate were obtained by using a Nanoscope VMM8Multimode microscope. A V2 TESPA tip was used, operating in tapping mode in air. *R*_a_ values were determined over a 5 μm × 5 μm square section.

### XPS

Spectra of inside and outside the wear scar of the additive **3** tribofilm on a steel plate were collected using a SPECS EnviroESCA near-ambient pressure XPS, which is equipped with a monochromatic Al K_α_ X-ray source (1486.71 eV) operated at 42 W. All measurements were performed under vacuum. High-resolution spectra for C 1s, V 2p, and O 1s were collected as an average of 4 scans with a step size of 0.1 eV, dwell time of 0.1 s, and pass energy of 50 eV. The binding energy scale was calibrated for surface charging using the narrow primary O 1s peak at 530.0 eV. Data was analyzed using CasaXPS. Shirley backgrounds and Gaussian–Lorentzian curves (60% Gaussian; 40% Lorentzian) were used to fit the spectra. Vanadium peaks were fit following literature precedent, with a constraint on the Δvalue between the V 2p_3/2_ and the O 1s components for each oxide species present.

### NEXAFS

Soft X-ray total electron yield V L_2,3_-edge (519.8 and 512.1 eV) and O K-edge (543.1 eV) NEXAFS spectra of the inside and outside of the wear scar of the additive **3** tribofilm on a steel plate were collected at the versatile soft X-ray (VERSOX) beamline B07-B (Diamond Light Source).^65^ All measurements were performed under a 1 mbar of helium environment. The photon energy scale was calibrated for beamline effects and surface charging using the K-edge at 530.0 eV. The data normalization procedure using a He gas phase spectrum is detailed elsewhere. Notably, the terms NEXAFS and XANES are synonymous, but for the sake of distinction, NEXAFS is used for soft X-ray XAS and XANES is for hard X-ray XAS.

### XANES

Hard X-ray fluorescence yield V K-edge (5465 eV) XANES spectra of the inside and outside of the wear scar of the additive **3** tribofilm on a steel plate and compound **3** as a powder were collected at the microfocus XAS beamline I18 (Diamond Light Source).^66^ The location of the wear scar on the steel plate was identified using scanning micro XRF mapping (Figure S13). Reference spectra of vanadium oxide powders (i.e., VO, V_2_O_3_, VO_2_, and V_2_O_5_) were collected in transmission mode at the core XAS beamline B18 (Diamond Light Source) and obtained from the beamline’s XAS data repository.^67,68^ All measurements were performed at room temperature under a constant helium environment.^69^ Background subtraction and normalization of the experimental XANES data were done using Athena.^70^

## Supporting Information Available

Synthetic procedures and characterization data for compounds **1**–**3**, detailed tribological data, and supporting tribofilm characterization data (PDF)
