# Supplementary material for: Green Alternatives to Zinc Dialkyldithiophosphates: Vanadium Oxide-Based Additives
Source: ACS Appl Eng Mater. 2023 Oct 31;1(11):2916–25. doi: 10.1021/acsaenm.3c00425 (PMC10682961; doi:10.1021/acsaenm.3c00425)
Supplement: Supplementary file 1 — em3c00425_si_001.pdf [file em3c00425_si_001.pdf]

*Supplementary Materials for

Green Alternatives to Zinc Dialkyldithiophosphates: Vanadium Oxide Based Additives

Andrew. J. Straiton,[a] Thokozile. A. Kathyola,*[b,c] Callum. Sweeney,[d] James D. Parish,[a,e] Elizabeth A. Willneff,[f] Sven. L. M. Schroeder,[b,c] Ardian Morina,[d] Anne Neville,[d] Joshua J. Smith,[e] Andrew L. Johnson,*[a]

a. Department of Chemistry, University of Bath, Claverton Down, Bath,BA2 7AY.
b. School of Chemical and Process Engineering, University of Leeds, Woodhouse Lane, Leeds, LS2 9JT.
c. Diamond Light Source., Harwell Science and Innovation Campus, Fermi Ave, Didcot, OX11 0DE.
d. School of Mechanical Engineering, University of Leeds, Woodhouse Lane, Leeds, LS2 9JT.
e. Infineum UK Ltd., Milton Hill Business and Technology Centre, Abingdon, Oxfordshire, OX13 6BB.
f. School of Design, University of Leeds, Woodhouse Lane, Leeds, LS2 9JT.

Correspondence to: a.l.johnson@bath.ac.uk and t.a.kathyola@diamond.ac.uk.

This PDF file includes:

Synthesis and Characterisation of Compounds **1** – **3**
Details of Tribological Testing
Figures S1 to S15
Tables S1 to S3
Supplementary References

Synthesis and Characterisation

1.1 Synthetic Procedures

3-n-Octyl-2,4-pentanedionate (C8-acacH):

Prepared via an adapted literature procedure using acetylacetone acetyl acetone (40.3 ml. 395.0 mmol) and 1-iodooctane (71.0 ml, 395.0 mmol).[1] The resulting oil was purified by column chromatography to give 41.0 g (49 %) of the product as a pale-yellow oil.

^{1}H NMR (500 MHz, C_6D_6) δ_H: 0.91 (3H, m, RCH_3), 1.03-1.28 (12H, m, CH_2***C_6H_{12}***CH_3), 1.64 (2H, m, ***CH_2***R-keto), 1.77 (6H, s, C(O)***CH_3***-keto), 1.82 (6H, s, C(O)***CH_3***-enol), 1.86 (2H, m, ***CH_2***R-enol), 3.24 (1H, t, J= 7 Hz, C(O)***CH***).
^{13}C{^{1}H} NMR (125.7 MHz, C_6D_6) δ_C: 14.0 (R***CH_3***), 22.2 (***CH_3***C(O)-enol), 22.69 (***R***), 22.71 (***R***), 27.3 (***R***), 27.5 (***CH_2***R}-enol), 28.0 (***CH_2***R}-keto), 28.1 (***CH_3***C(O)-keto), 29.3 (***R***), 29.3 (***R***), 29.4 (***R***), 29.5 (***R***), 30.6 (***R***), 31.8 (***R***), 31.9 (***R***), 68.6 (***C***H(O)-keto), 110.2 (***C***C(O)-enol), 190.5 (***C***(O)-enol), 202.7 (***C***(O)-keto).
HRMS (ESI+): calculated for M+ $C_{13}H_{24}O_2$, 212.1776; found, 212.1779.

Elemental Analysis: Found (Calculated) C: 74.1 (73.5), H: 11.5 (11.4).

TiO(C8-acac)$_2$ (1):

To a cooled solution of $Ti(O^iPr)_4$ (5.68 g, 20.0mol) in Et_2O/Hexane (20 mL, 20 mL) was added 3-n-Octyl-2,4-pentanedionate (8.49 g, 40.0 mmol) dropwise. In a separate flask, Et_2O (20 mL) and H_2O (0.360 ml, 20.0 mmol) were stirred and added dropwise to the cooled solution of $Ti(O^iPr)_4$. Volatiles were removed *in vacuo* after 30 minutes to yield a yellow oil. Small quantities of crystals suitable for single-crystal X-ray diffraction were obtained through recrystallisation from hexane at -28 °C. The bulk of the product was 7.78 g, (80 %) of yellow oil, determined by NMR to be a mixture of isomers in line with literature precedent.[2]

Single-crystal X-ray diffraction data can be found in Figure S1 and Table S1
^{1}H NMR, 500 MHz, C_6D_6: 0.923 (3H, t, J=7 Hz, ***Alkyl***), 1.22-1.32 (10H, m, ***Alkyl***), 1.65 (2H, m, ***Alkyl***), 1.99 (6H, s, C(O)***CH_3***), 2.02 (2H, m, C***CH_2***Alkyl).
^{13}C NMR, 125 MHz, C_6D_6: 14.0 (R***CH_3***), 22.8 (***Alkyl***), 24.6 (C(O)***CH_3***), 28.1 (***Alkyl***), 29.3 (***Alkyl***), 29.4 (***Alkyl***), 29.5(***Alkyl***), 29.8 (***Alkyl***), 30.5 (***Alkyl***), 32.0 (C***CH_2***Alkyl), 68.7 (***C***C(O)), 113.6 (***C***C(O)), 190.8 (***C***O), 203.1 (***C***O).

Elemental Analysis: Found (Calculated) C: 64.42 (64.19), H: 9.56 (9.53).

Zr(C8-acac)$_4$ (2):

To a cooled solution of $Zr(NMe_2)_4$ (5.35 g, 20.0 mmol) in hexane (100 mL) was added 3-n-Octyl-2,4-pentanedionate (17.0, 80.0 mmol) dropwise. Volatiles were removed *in vacuo* after 30 minutes to yield a yellow/orange oil, which was recrystallised to give 15.7 g (83 %) of yellow orange oil as the product.

^{1}H NMR, 500 MHz, C_6D_6: 0.90 (3H, t, J=7 Hz, R***CH_3***), 1.25 (m, ***Alkyl***), 2.06 (6H, s, CO***Me***), 2.15 (2H, t, J=7 Hz, ***CH_2***Alkyl)

^{13}C NMR, 125 MHz, C_6D_6: 14.4 (alkyl***CH$_3$***) , 23.1 (***R***), 25.6 (CO***CH$_3$***), 29.7 (***R***), 29.8 (***R***), 29.9 (***R***), 30.3 (***R***), 31.8 (***R***), 32.3 (***R***), 112.3 ({OC}$_2$***C***R), 187.0 (***C***=O)

Elemental Analysis: Found (Calculated) C: 66.72 (66.69) H: 9.73 (9.90) N: 0.00 (0.00)

VO(C8-acac)$_2$ (3):

C8-acac (8.49 g, 17.02 mmol) was added to $VOSO_4$ (5.06 g, 20.0 mmol) and NaOAc,(4.10 g, 50 mmol) suspended EtOH (40 ml) under an N_2 atmosphere. Whilst remaining under an inert atmosphere, water (20 ml) was added and the reaction left to stir for 5 minutes, in which time a green oil began to partition. A further portion of water (25 ml) was subsequently added to ensure full precipitation of the oil, which, upon scratching the walls of the flask and upon cooling to 0 °C turned into a solid precipitate. The reaction was stirred for a further 2 hours following which the solid was isolated via filtration, and washed with water (3 x 10 ml), cold ethanol (20 ml) and heptane (3 x 5ml). The resulting solid was dried under vacuum, giving 5.75 g (58 %) of green powder. crystals suitable for single-crystal X-ray diffraction were obtained through recrystallisation from hexane at -28 °C, which confirmed the structure of the product, and is in line with that reported previously.[1] Characterisation via NMR is not possible as the compound is paramagnetic.

μ_{eff} = 1.56
IR ν_{max} / cm^{-1}: 997 (*V=O*), 1290, 1325, 1466, 1557, 2870, 2956, 2961

Elemental Analysis: Found (Calculated) C: 63.40 (63.79) H: 9.66 (9.47) N: 0.00 (0.00)

1.2 Chemical Characterisation

^1H and ^{13}C NMR spectra were recorded on Bruker Advance 400 or 500 MHz FT-NMR spectrometers in saturated solutions of $CDCl_3$ and C_6D_6 at room temperature. Chemical shifts are expressed in ppm with respect to Me_4Si (^1H and ^{13}C). Magnetic moments were determined using Evans method.[3–5] IR spectra were recorded on a Perkin Elmer Spectrum 100 ATR FT-IR Spectrometer whilst high resolution mass spectrometry results were acquired on an externally calibrated Agilent QTOF UHR-ToF mass spectrometer coupled to an electrospray source (ESI-QToF). Thermogravimetric analysis was undertaken on a PerkinElmer TGA 4000 under a flow of Ar (20 mL min^{-1}) ramping at 5 °C min^{-1}.

Single crystal X-ray diffraction data was collected at 150 K using either an Agilent Xcalibur or Agilent SuperNova Dual diffractometer with either Mo-Kα (λ = 0.71073 Å) or Cu-Kα (λ = 1.5418 Å) radiation. Structures were solved by fill-matrix least squares refinement using either the WinGX-170 suite of programs or the X-SEED programme suite.

1.3 Preparation of Oils

A 4 cSt group III mineral oil and a model ZDDP were provided by Infineum UK Ltd. Fully formulated oils used in this study are commercially representative model systems, with analytics as presented in table S3. Lubricant samples for testing were prepared by blending 10 mmol of additive into either base oil, FFO1 or FFO3 at 60 °C with stirring. ZDDP and MoDTC in the model samples were at commercially representative concentrations.

1.4 Tribological Testing

The lambda value (λ) was determined using equation S1.1.

$$\lambda = \frac{h_{min}}{\sigma^*} \#(1.1)$$

Where:

$$\sigma^* = \sqrt{\sigma_1^2 + \sigma_2^2} \#(1.2)$$

And the minimum film thickness can be given by:

$$\frac{h_{min}}{R_x} = 3.63\left(\frac{U\eta_0}{E^* R_x}\right)^{0.68} (\alpha E^*)^{0.49}\left(\frac{W}{E^* R_x^2}\right)^{-0.073} (1 - e^{-0.68k}) \#(1.3)$$

Where R_x is the radius of curvature in the *x* direction, *U* is the entrainment speed, *E** is the reduced Young's Modulus, η_0 is the dynamic viscosity of the lubricant, α is the pressure-viscosity coefficient, *W* is the applied load and *k* = 1. The entrainment speed is given by the following:

$$U = \frac{U_1 + U_2}{2} \#(1.4)$$

The reduced Young's Modulus is given by:

$$\frac{1}{E^*} = \frac{1 - \nu_1^2}{E_1} + \frac{1 - \nu_2^2}{E_2} \#(1.5)$$

Where υ_1 and υ_2 are the Poisson's Ratio of each material and E_1 and E_2 are the Young's Modulus of each material.

1.5 Wear Measurements

The worn volume of the pins has been measured via 3D white light interferometry profilometry, using a non-contact NPFLEX.

The worn volumes were converted into dimensionless wear coefficients, K, *via* Archard's equation:

$$K = \frac{3HV}{PL} \#(1.6)$$

Where V is the worn volume (mm^3), H is the Brinell Hardness of the sample (7450 Nmm^{-2}), P is the normal load (50 N) and L is the total length covered by the reciprocating pin (1.26 $x10^3$ m).

A)

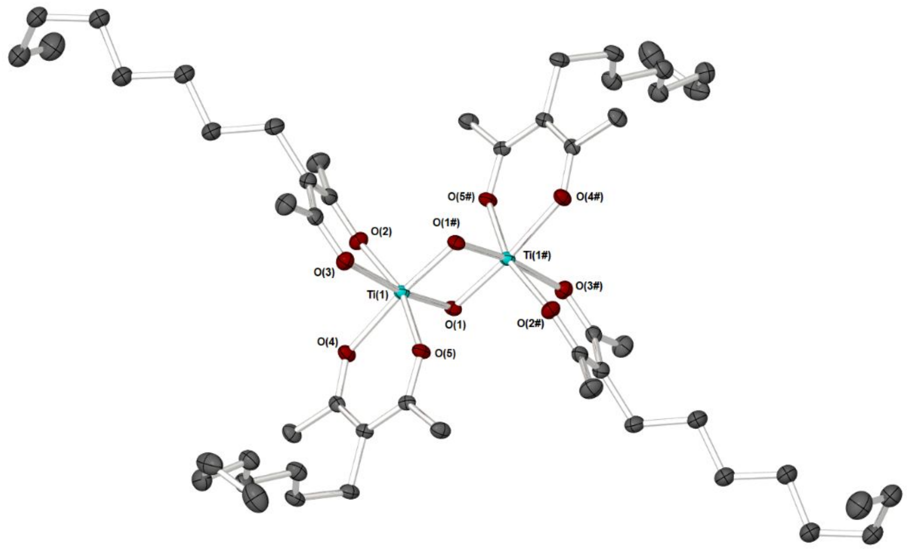

B)

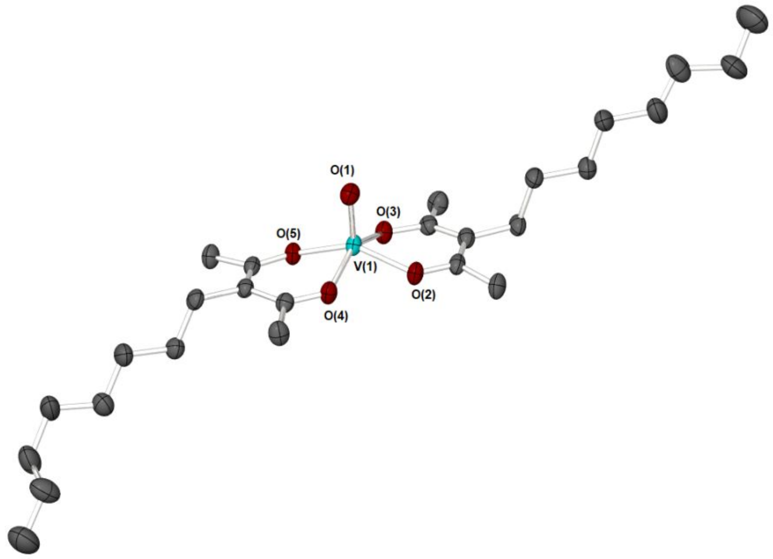

Figure S1. Molecular structure of a) TiO(C8-acac)$_2$, (**1**) and b) VO(C8-acac)$_2$ (**3**), previously reported by Preininger et al.[1] Hydrogen atoms have been omitted for clarity. Thermal ellipsoids shown at 50% probability. Crystallographic data contained in Table S1.

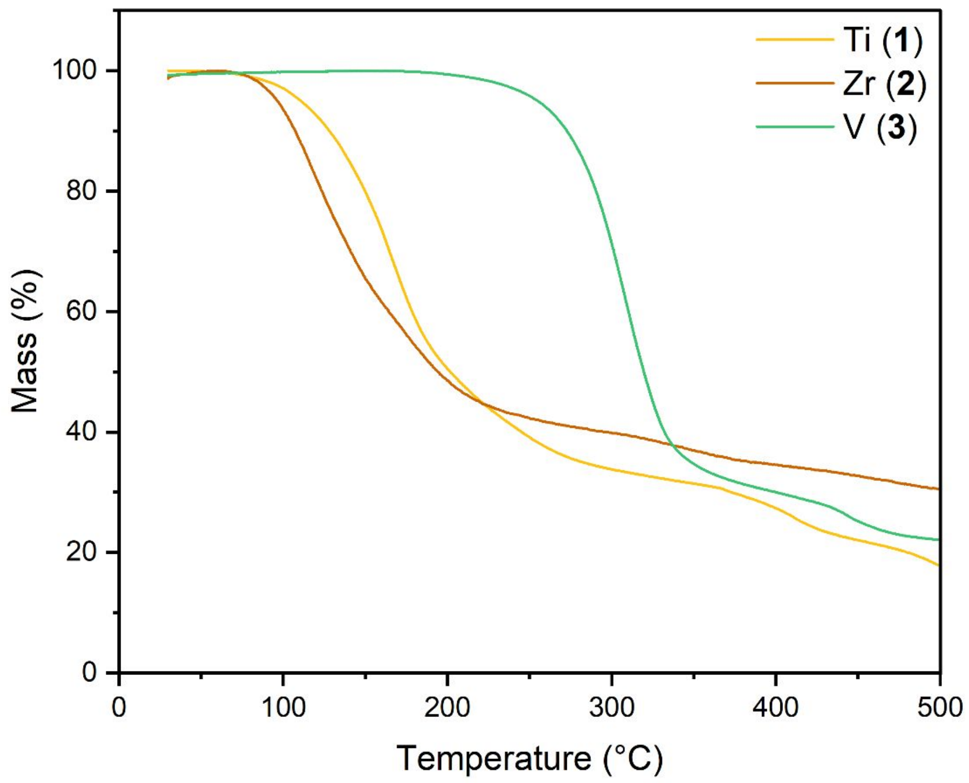

Figure S2. Thermogravimetric plot for compounds 1-3.

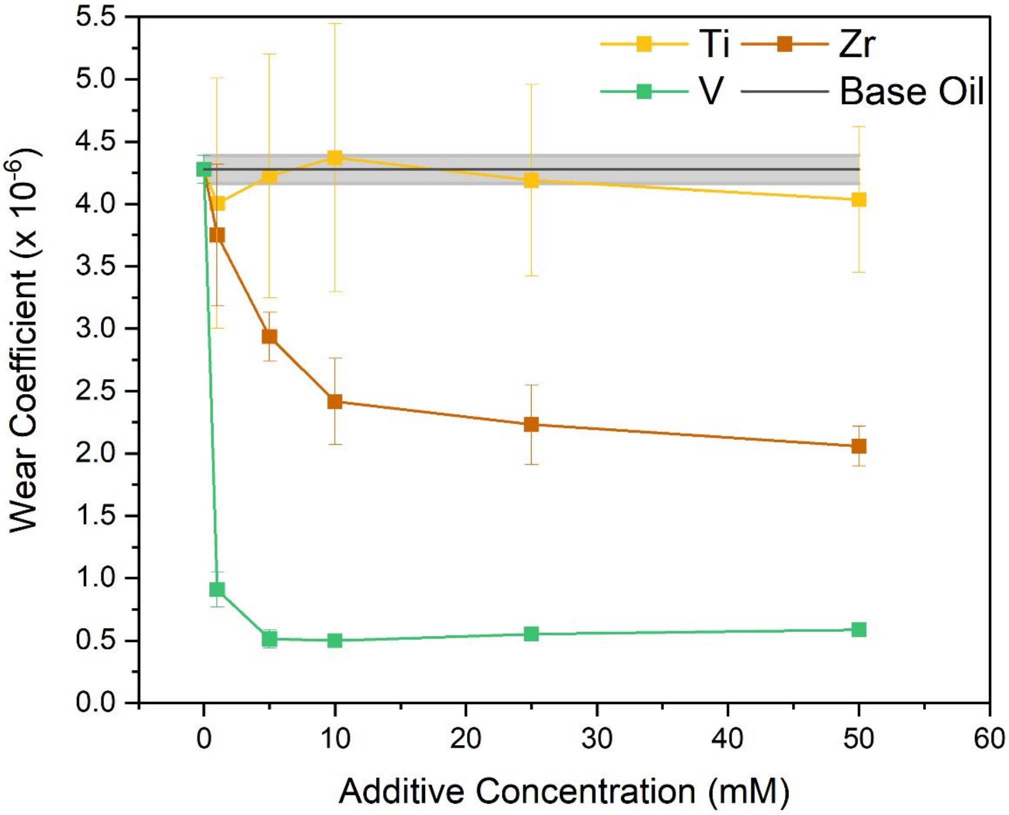

Figure S3. Wear coefficient as a function of additive concentration for compounds **1**-**3** in base oil. Data collected using TE 77 reciprocating pin-on-plate tests of each additive at a contact pressure of 250 MPa. Corresponding coefficient of friction measurements displayed in Figure S6. Data for base mineral oil is provided as reference.

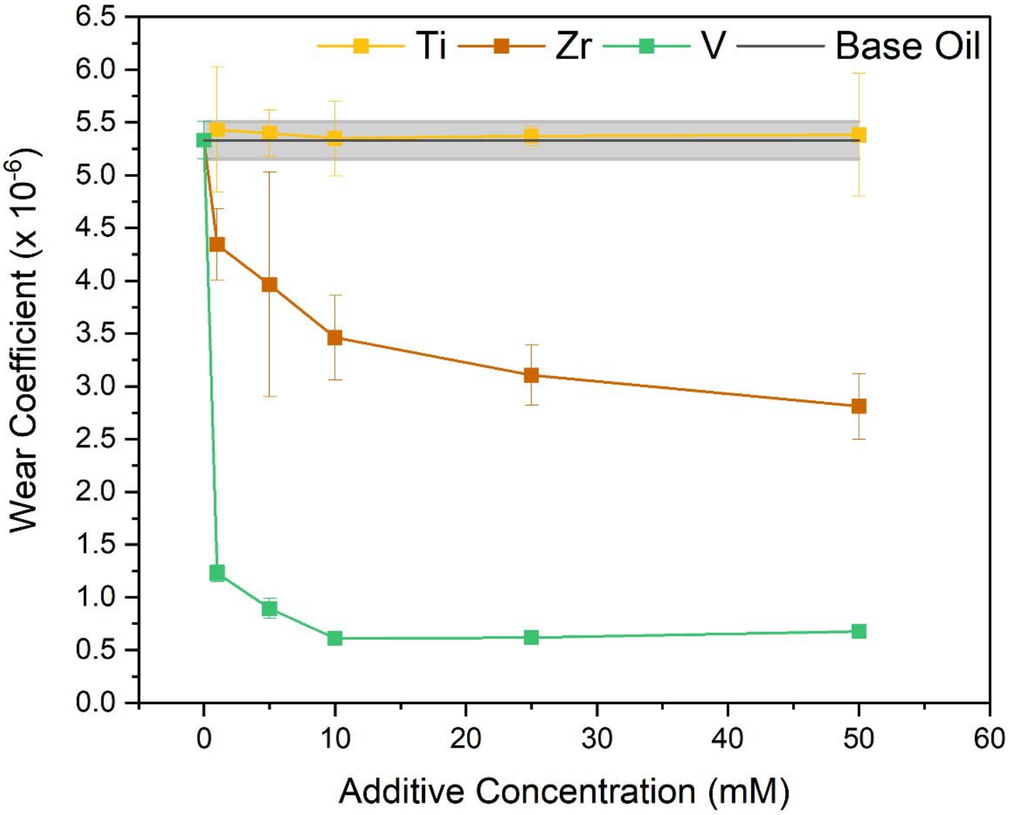

Figure S4. Wear coefficient as a function of additive concentration for compounds **1**-**3** in base oil. Data collected using TE 77 reciprocating pin-on-plate tests of each additive at a contact pressure of 1 GPa. Corresponding coefficient of friction measurements displayed in Figure S7. Data for base mineral oil is provided as reference.

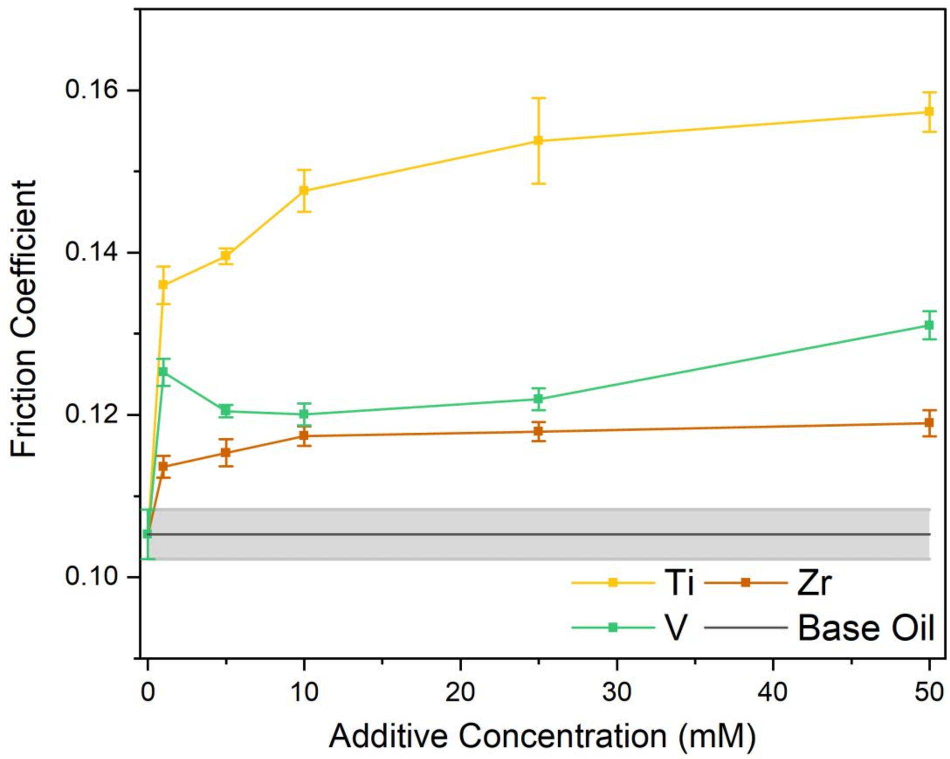

Figure S5. Coefficient of friction as a function of additive concentration for compounds **1**-**3** in base oil. Data collected using TE 77 reciprocating pin-on-plate tests of each additive at a contact pressure of 250 MPa. Corresponding wear coefficient measurements displayed in Figure S4. Data for base oil is provided as reference.

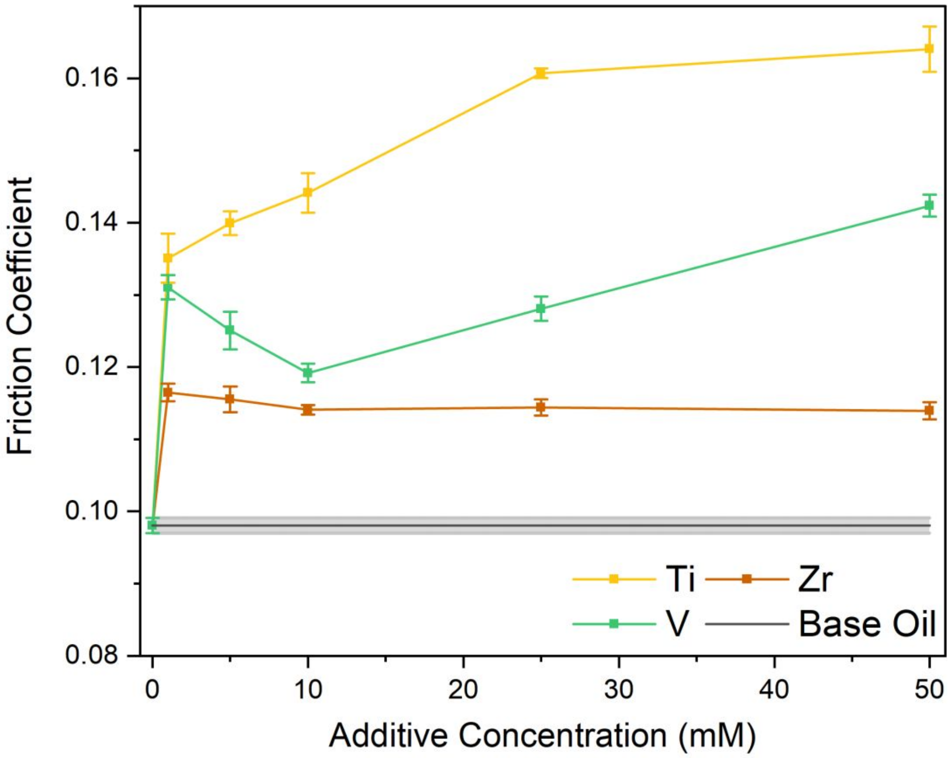

Figure S6. Coefficient of friction as a function of additive concentration for compounds **1**-**3** in base oil. Data collected using TE 77 reciprocating pin-on-plate tests of each additive at a contact pressure of 1 GPa. Corresponding wear coefficient measurements displayed in Figure S5. Data for base oil is provided as reference.

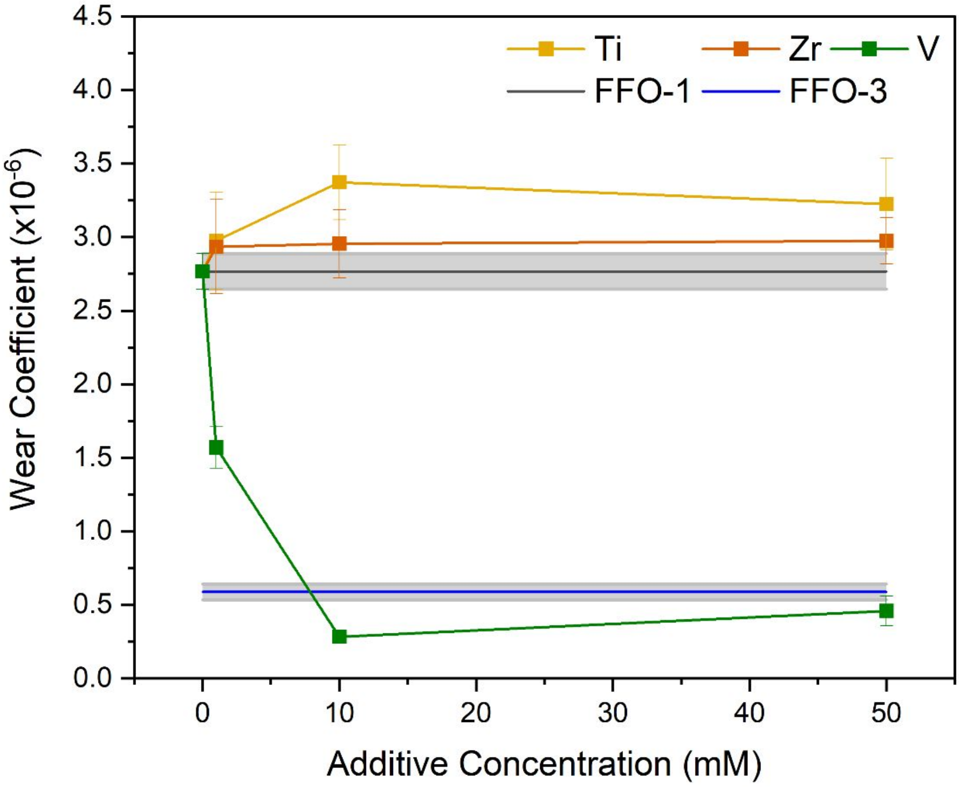

Figure S7. Wear coefficient as a function of additive concentration for compounds **1**-**3** in a commercially representative oil without ZDDP and MoDTC loading (FFO-1). Data collected using TE 77 reciprocating pin-on-plate tests of each additive at a contact pressure of 250 MPa. Corresponding coefficient of friction measurements displayed in Figure S10. Data for commercially representative oils FFO-1 and FFO-3, defined in Table S3, are provided as references.

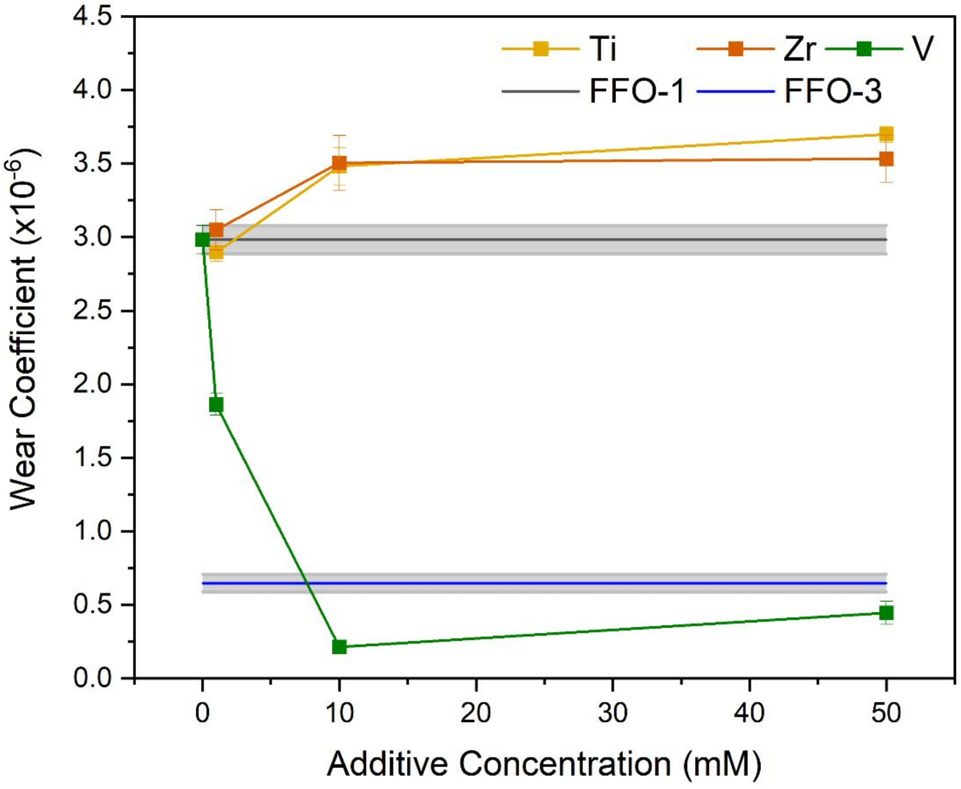

Figure S8. Wear coefficient as a function of additive concentration for compounds **1**-**3** in a commercially representative oil without ZDDP and MoDTC loading (FFO-1). Data collected using TE 77 reciprocating pin-on-plate tests of each additive at a contact pressure of 1 GPa. Corresponding coefficient of friction measurements displayed in Figure S11. Data for commercially representative oils FFO-1 and FFO-3, defined in Table S3, are provided as references.

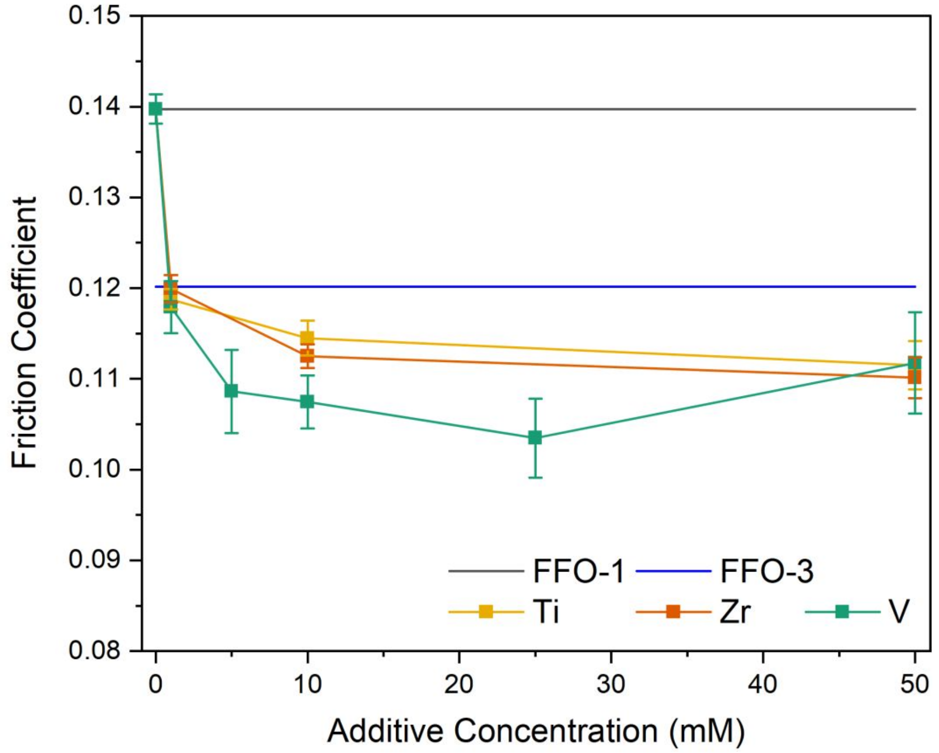

Figure S9. Coefficient of friction as a function of additive concentration for compounds **1**-**3** in commercially representative oil without ZDDP and MoDTC loading (FFO-1). Data collected using TE 77 reciprocating pin-on-plate tests of each additive at a contact pressure of 250 MPa. Corresponding wear coefficient measurements displayed in Figure S8. Data for commercially representative oils FFO-1 and FFO-3, defined in Table S3, are provided as references.

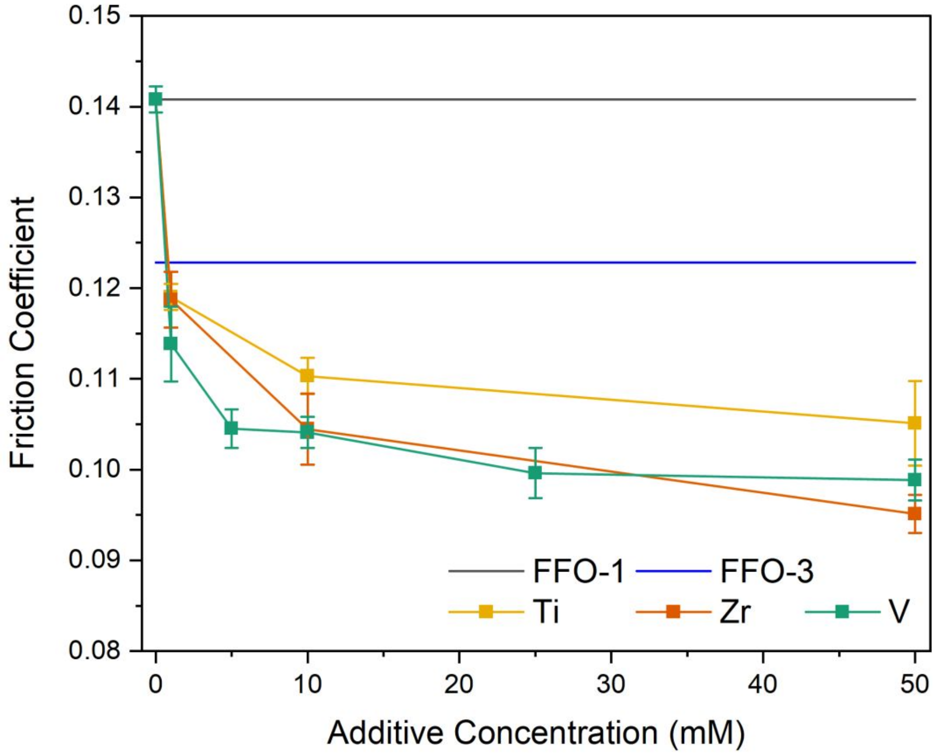

Figure S10. Coefficient of friction as a function of additive concentration for compounds **1**-**3** in commercially representative oil without ZDDP and MoDTC loading (FFO-1). Data collected using TE 77 reciprocating pin-on-plate tests of each additive at a contact pressure of 1 GPa. Corresponding wear coefficient measurements displayed in Figure S9. Data for commercially representative oils FFO-1 and FFO-3, defined in Table S3, are provided as references.

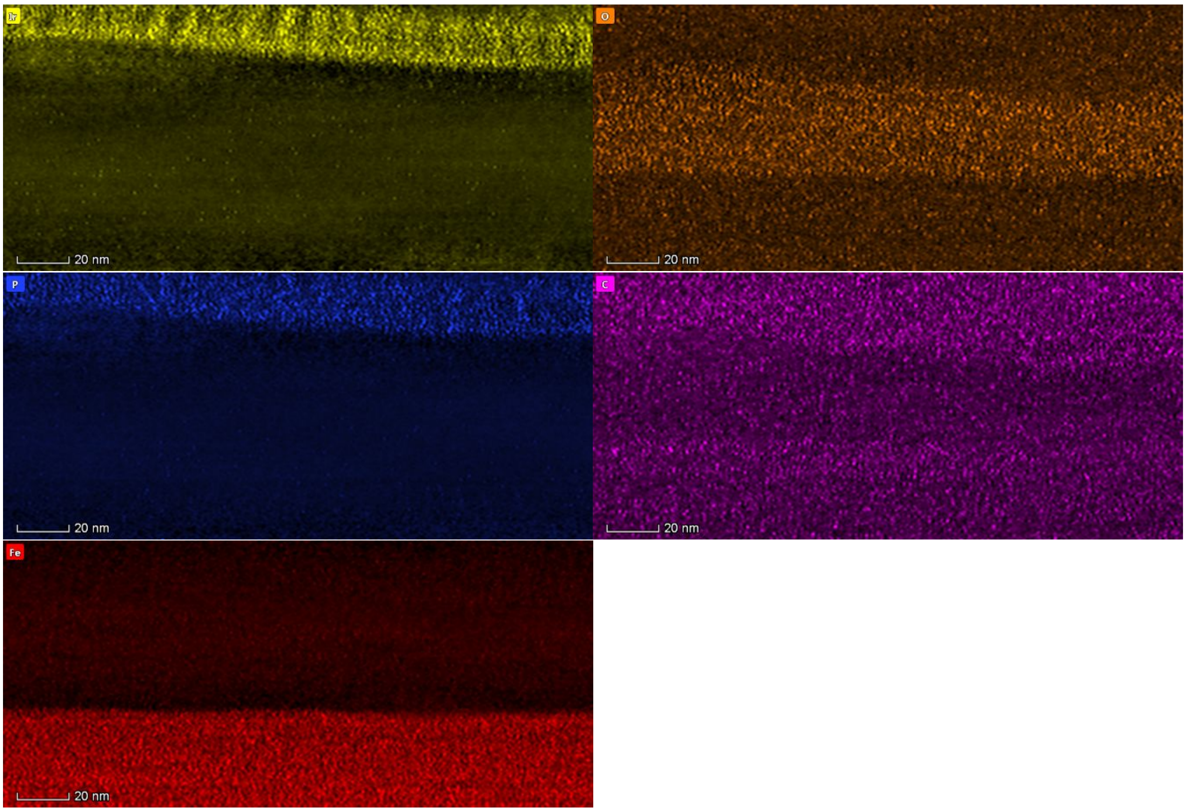

Fig S11. EDX images of the cross-section of a tribofilm formed from **3**. A TEM image of the corresponding tribofilm is in figure 4.

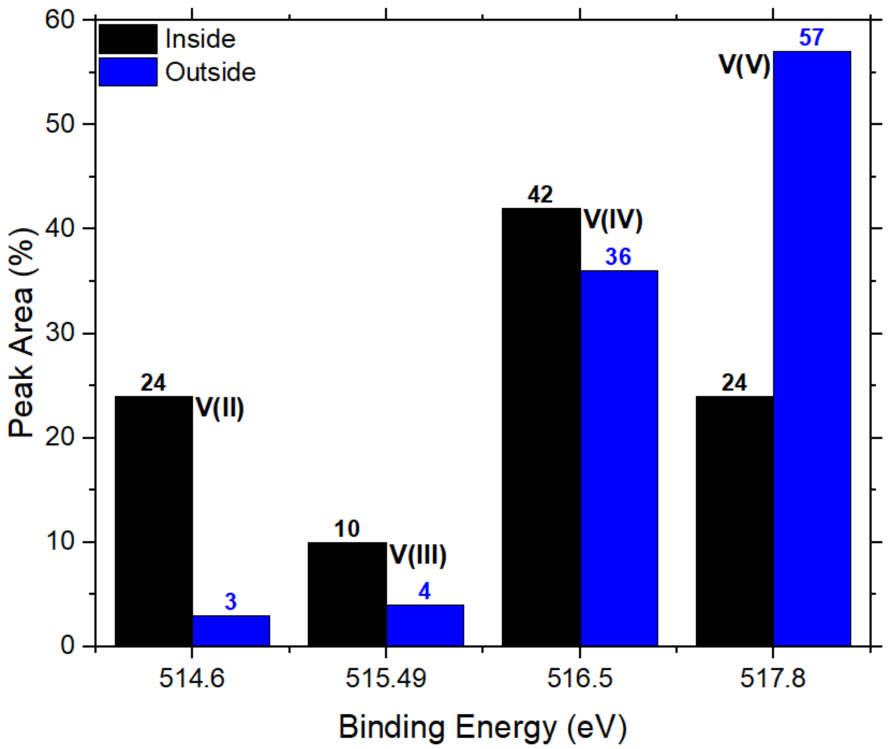

Figure S12. Comparison of the relative oxidation state contributions inside and outside the wear scar of a tribofilm formed from **3**, as determined by XPS.

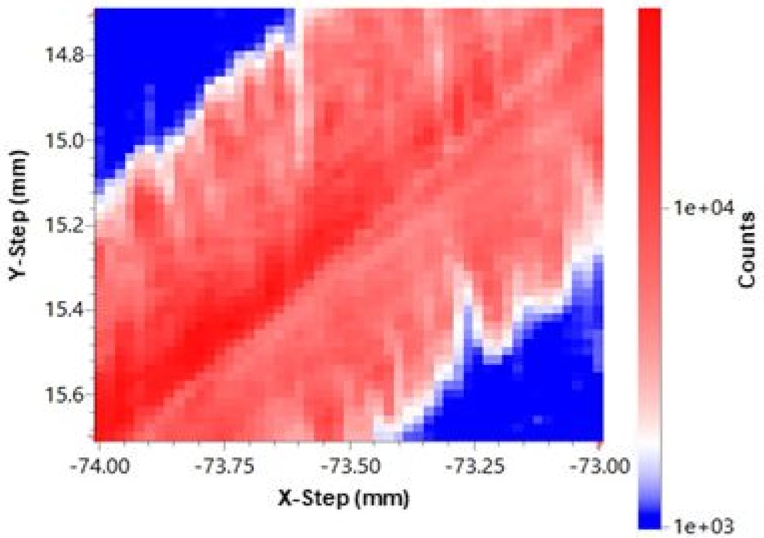

Figure S13. Scanning X-ray fluorescence (XRF) map of the wear scar used to identify the wear scar location by virtue of the high localised concentration of vanadium. Subsequent measurements "inside" the wear scar are obtained within the red region, and "outside" in the blue region.

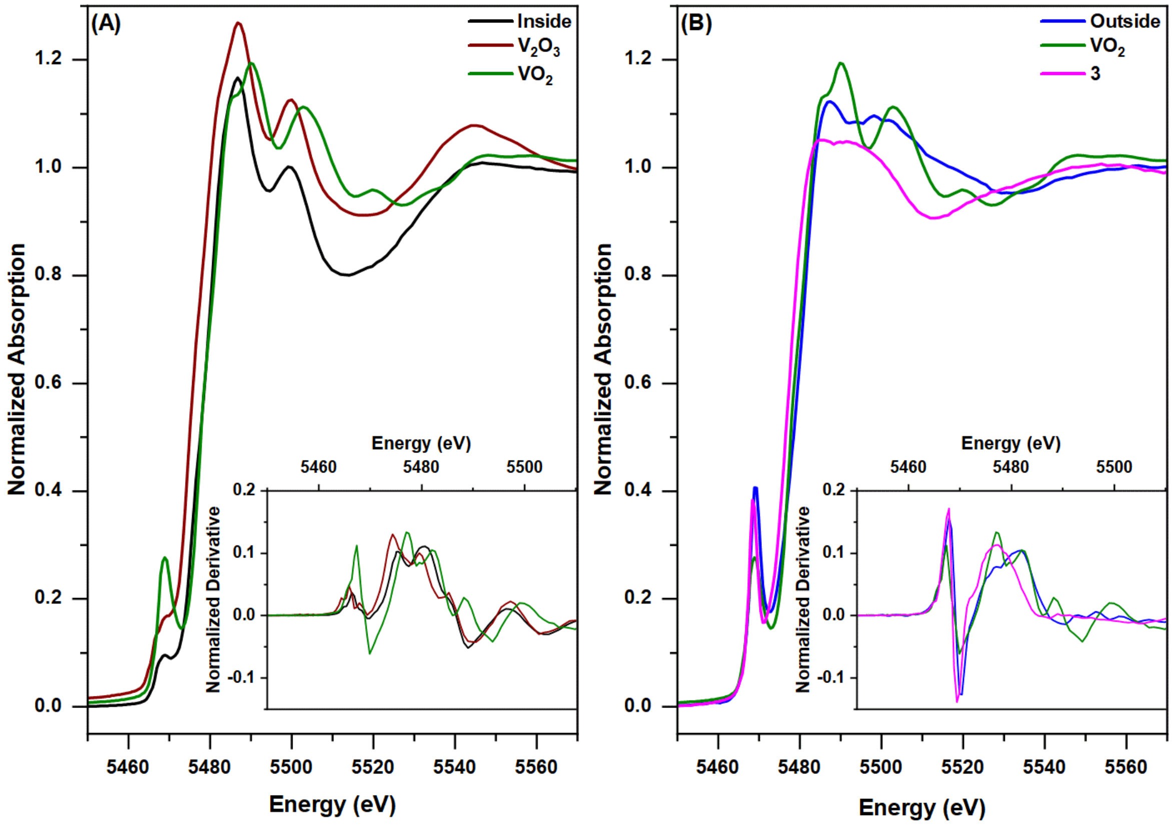

Figure S14. XANES spectra from A) inside and B) outside the wear scar, overlayed with relevant vanadium oxide reference data. Inset: Derivatives of the XANES spectra.

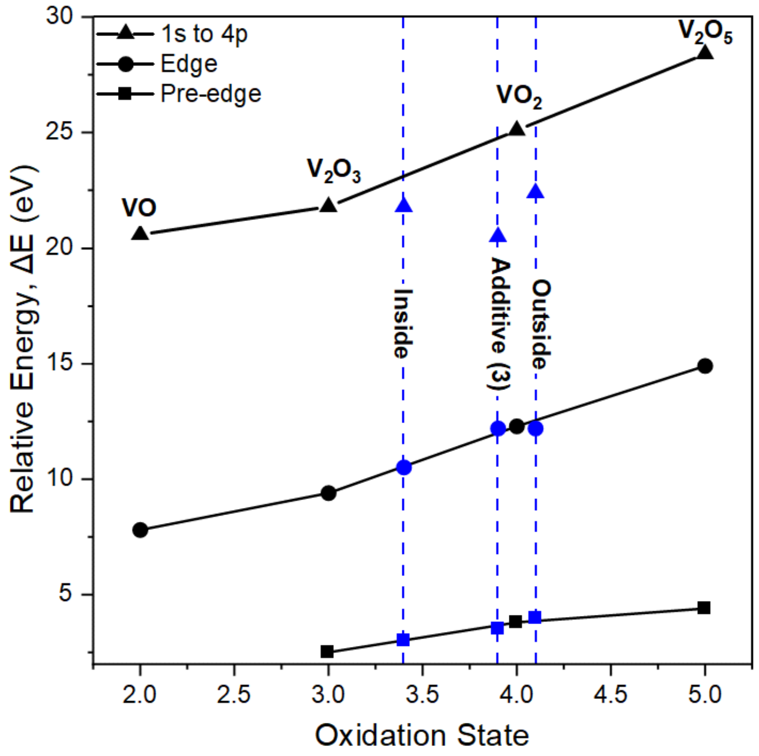

Figure S15. Comparison of the vanadium oxide peak positions inside and outside the wear scar with relevant vanadium oxide references. See Figure S13 for the X-ray fluorescence map used to identify areas inside and outside the wear scar.

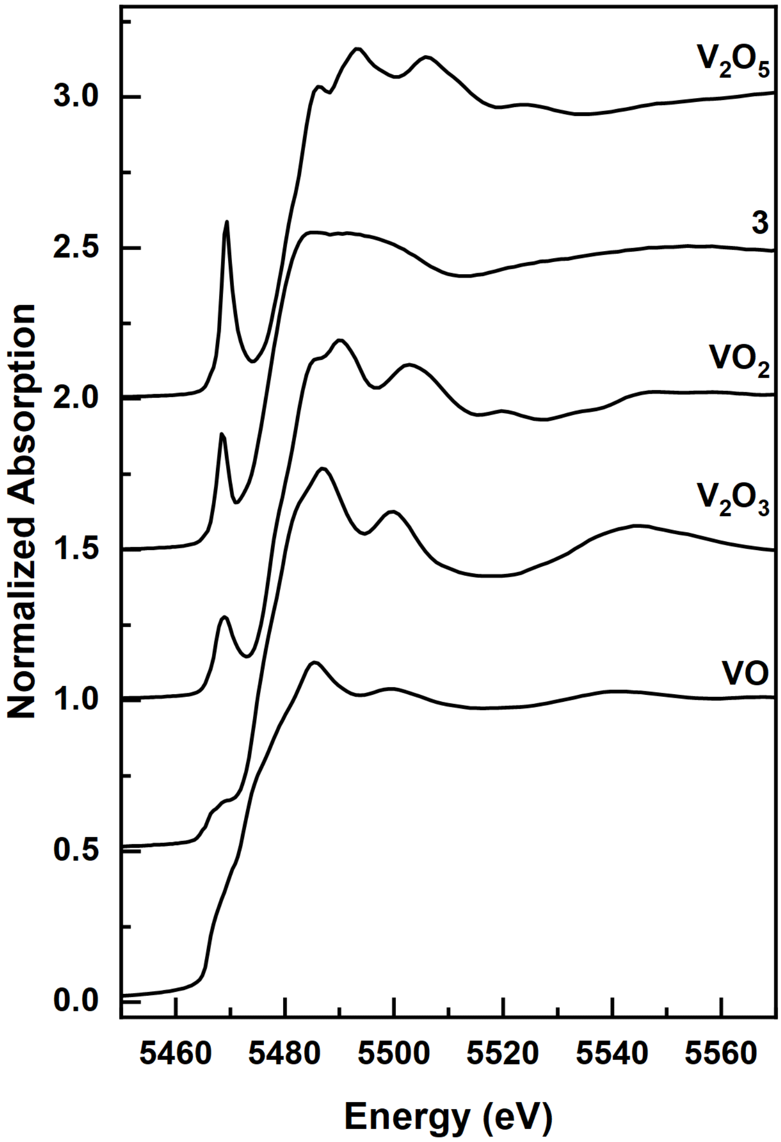

Figure S16. The XANES spectra of multiple relevant vanadium oxide standards, including that of compound **3**.

Table S1. Crystallographic data for $TiO(C8\text{-}acac)_2$ (**1**) and $VO(C8\text{-}acac)_2$ (**3**).

	1	3
**Empirical formula**	C52 H92 O10 Ti2	C26 H46 O5 V
**Formula weight**	973.05	489.57
**Temperature**	150.00(10) K	150.00(10) K
**Wavelength**	1.54184 Å	1.54184 Å
**Crystal system**	Triclinic	Monoclinic
**Space group**	P-1	$P2_1/c$
**Unit cell dimensions**	a = 7.92610(10) Å a= 100.2630(10)°.<br>b = 9.3142(2) Å b= 93.4450(10)°.<br>c = 19.1192(3) Å g = 107.656(2)°.	a = 15.5530(2) Å a= 90°.<br>b = 8.46827(10) Å b= 97.0119(14)°.<br>c = 21.3781(3) Å g = 90°.
**Volume**	1313.63(4) Å3	2794.59(6) Å3
**Z**	1	4
**Density (calculated)**	1.230 Mg/m3	1.164 Mg/m3
**Absorption coefficient**	3.013 mm-1	3.204 mm-1
**F(000)**	528	1060
**Crystal size**	0.339 x 0.184 x 0.026 mm3	0.308 x 0.244 x 0.090 mm3
**Theta range for data collection**	4.736 to 73.403°.	2.863 to 73.097°.
**Index ranges**	-8<=h<=9, -11<=k<=11, -23<=l<=23	-18<=h<=12, -10<=k<=10, -26<=l<=26
**Reflections collected**	38849	22225
**Independent reflections**	5243 [R(int) = 0.0419]	5524 [R(int) = 0.0338]
**Completeness to theta = 67.684°**	99.90%	100.00%
**Absorption correction**	Gaussian	Gaussian
**Max. and min. transmission**	1.000 and 0.353	0.956 and 0.480
**Refinement method**	Full-matrix least-squares on F2	Full-matrix least-squares on F2
**Data / restraints / parameters**	5243 / 0 / 295	5524 / 12 / 343
**Goodness-of-fit on F2**	1.051	1.025
**Final R indices [I>2sigma(I)]**	R1 = 0.0310, wR2 = 0.0864	R1 = 0.0360, wR2 = 0.0971
**R indices (all data)**	R1 = 0.0321, wR2 = 0.0873	R1 = 0.0402, wR2 = 0.1013
**Extinction coefficient**	n/a	n/a
**Largest diff. peak and hole**	0.421 and -0.295 e.Å-3	0.213 and -0.335 e.Å-3

Table S2. Details of the commercially representative model oils used in tribological testing.

	FFO-1	FFO-2	FFO-3
**Elemental Analysis**			
**Mo**	X	X	24
**P / ppm**	X	765	765
**Zn / ppm**	X	830	830
**Viscometrics**			
**SAE Grade**	5W-30	5W-30	5W-30

Table S3. Peak fitting data for XPS studies on a tribofilm formed during tribological testing of **3**

Peak			Component	Binding Energy	FWHM	%Area
**V**	**2p**	**J=3/2**	V(V)	517.3	2.30	15%
			V(IV)	515.8	2.81	7%
			V(III)	515.3	2.81	79%
		**J=1/2**	V(V)	524.7	2.36	15%
			V(IV)	523.2	2.98	7%
			V(III)	522.7	3.32	79%
**O**	**1s**		1	529.8	1.32	58%
			2	531.3	2.00	38%
			3	533.3	2.00	3%
**C**	**1s**		C-C,C-H	284.8	1.46	71%
			C-OH, C-O-C	286.3	1.46	15%
			C=O	287.8	1.46	8%
			O-C=O	288.8	1.46	6%

Supplementary References

1 O. Preininger, I. Charamzová, J. Vinklárek, I. Císařová and J. Honzíček, Oxovanadium(IV) complexes bearing substituted pentane-2,4-dionate ligands: Synthesis, structure and drying activity in solvent-borne alkyd paints, *Inorganica Chim. Acta*, 2017, **462**, 16–22.

2 M. Puchberger, W. Rupp, U. Bauer and U. Schubert, Reaction of metal alkoxides with 3-alkyl-substituted acetylacetone derivatives - Coordination vs. hydrodeacylation, *New J. Chem.*, 2004, **28**, 1289–1294.

3 D. F. Evans, The determination of the paramagnetic susceptibility of substances in solution by nuclear magnetic resonance, *J. Chem. Soc.*, 1959, **81**, 2003–2005.

4 E. M. Schubert, Utilizing the evans method with a superconducting NMR spectrometer in the undergraduate laboratory, *Journol Chem. Educ.*, 1992, **69**, 62.

5 G. A. Bain and J. F. Berry, Diamagnetic corrections and Pascal's constants, *J. Chem. Educ.*, 2008, **85**, 532–536.
